# The protection of *Salicornia rubra* from ultraviolet radiation by betacyanins and phenolic compounds

**DOI:** 10.1002/pei3.10061

**Published:** 2021-09-23

**Authors:** Katherine Jensen, Roger T. Koide

**Affiliations:** ^1^ Department of Biology Brigham Young University Provo Utah USA

**Keywords:** Amaranthaceae, betacyanins, Caryophyllales, ecophysiology, phenolics, salt playa, ultraviolet radiation

## Abstract

*Salicornia rubra* is a commonly occurring annual species of the salt playas of the Great Basin Desert of the western United States. In such habitats, plants experience high levels of ultraviolet radiation, which could potentially damage DNA. As a member of the Amaranthaceae (Caryophyllales), *S*. *rubra* shoots typically contain high concentrations of the red‐violet pigments called betacyanins, which are ultraviolet‐absorbing compounds. Nevertheless, some specimens of *S*. *rubra* are green even when growing with full exposure to the sun. We, therefore, tested several hypotheses regarding the causes of variation among *S*. *rubra* plants in betacyanin concentration and the role of betacyanins in the absorption of ultraviolet radiation. We measured ultraviolet radiation absorption and the concentrations of betacyanins and phenolic compounds of the cell sap expressed from red and green plants growing in full sun, as well as plants grown under various levels of shade. We found that while betacyanin concentrations were predictable from plant color (red plants contained more betacyanins than green plants), the ability to absorb ultraviolet radiation was determined primarily by the concentration of phenolic compounds, which was determined by the level of exposure to the sun. Therefore, the DNA of green plants growing in full sun appears to be at no greater risk than the DNA of red plants.

## Graphical abstract

CAPTION: It shows the site of our research (Jensen and Koide), a portion of the salt playa near Goshen, UT, with its distinct vegetation zonation. The central band of red consists of *Salicornia rubra*. To the right is a yellow band of irregular thickness comprising *Sarcocornia utahensis*. To the right of that, the orange‐yellow grading to olive vegetation consists entirely of *Allenrolfea occidentalis*. The white material amidst the *Sarcocornia utahensis* and *Allenrolfea occidentalis* plants is surface salt.

Credit: Todd Jackson in the autumn of 2020

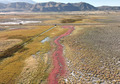

## 1 INTRODUCTION

*Salicornia rubra* A. Nelson is an annual, halophilic, stem‐succulent plant species commonly found in salt playas (Russell, 1883) of the Great Basin Desert of the United States (Khan et al., 2001; Weber, 2016). The floor of the Great Basin occurs at an elevation of approximately 1400 m above mean sea level, which increases the intensity of ultraviolet radiation compared with sea level (Blumthaler et al., 1997). Ultraviolet radiation can damage DNA directly by disrupting the phosphodiester backbone of DNA (Sinha & Häder, 2002), and indirectly by the generation of reactive oxygen species, which leads to DNA fragmentation via oxidative stress (de Jager et al., 2017). Salt playas are frequently occupied by shallow lakes in the spring that evaporate in the summer, leaving white evaporites, mainly NaCl, on the surface and in the upper layers of the soil (Khan et al., 2001). Thus, in addition to high levels of ultraviolet radiation, these plants are also subject to the separate stresses of high concentrations of NaCl and low water potentials (Tiku, 1976), which may further contribute to the production of reactive oxygen species by plants (Ahanger et al., 2017).

As a member of the Amaranthaceae (Caryophyllales), the red‐violet pigments produced by the shoots of *S*. *rubra* are nitrogen‐containing, non‐flavonoid betacyanins (Polturak & Aharoni, 2018). In the context of attracting particular pollinators using color, betacyanins apparently have the same functions as the more widely distributed anthocyanins, but the two types of compounds are never found in the same plant species (Stafford, 1994). Other than producing color, the roles of betacyanins in the ecology of plants have not been frequently investigated. However, it seems possible that betacyanins ameliorate some of the major environmental stresses experienced by *S*. *rubra* because they are both natural antioxidants (Sarker & Oba, 2019) and ultraviolet‐absorbing compounds (Sá et al., 2016). Indeed, betacyanins are assumed to be ecologically relevant to the protection of plants against ultraviolet radiation because their concentration in shoots may increase in response to increased ultraviolet radiation (Sá et al., 2016). Moreover, we have frequently observed that while the majority of *S*. *rubra* shoots at our research site are deep red, if some plants are shaded (by dead vegetation from the previous year’s growth, for example), they are frequently green as if, in an environment of low solar radiation, high concentrations of betacyanins are unnecessary. However, we also frequently observe green *S*. *rubra* plants occurring in full sun at our research site, which suggests that the red betacyanins are not the only means employed by *S*. *rubra* of absorbing ultraviolet radiation. Therefore, we have tested the following hypotheses concerning both the causes of variation among plants in betacyanin concentration and the role of betacyanins in the absorption of ultraviolet radiation:

1. Betacyanins absorb ultraviolet radiation.
2.
  a. Betacyanin concentrations are significantly greater in red plants than in green plants.
  b. Betacyanin concentrations are determined by exposure to sunlight.
3.
  a. Absorption of ultraviolet radiation is significantly greater in red plants than in green plants.
  b. Absorption of ultraviolet radiation is determined by exposure to sunlight.
4. Compounds other than betacyanins, such as phenolic compounds, absorb significant amounts of ultraviolet radiation.
5.
  a. Phenolic concentrations are significantly greater in green plants than in red plants.
  b. Phenolic concentrations are determined by exposure to sunlight.
6. Phenolic compounds are more important than betacyanins in absorbing ultraviolet radiation.

## 2 MATERIALS AND METHODS

**FIGURE 1:**
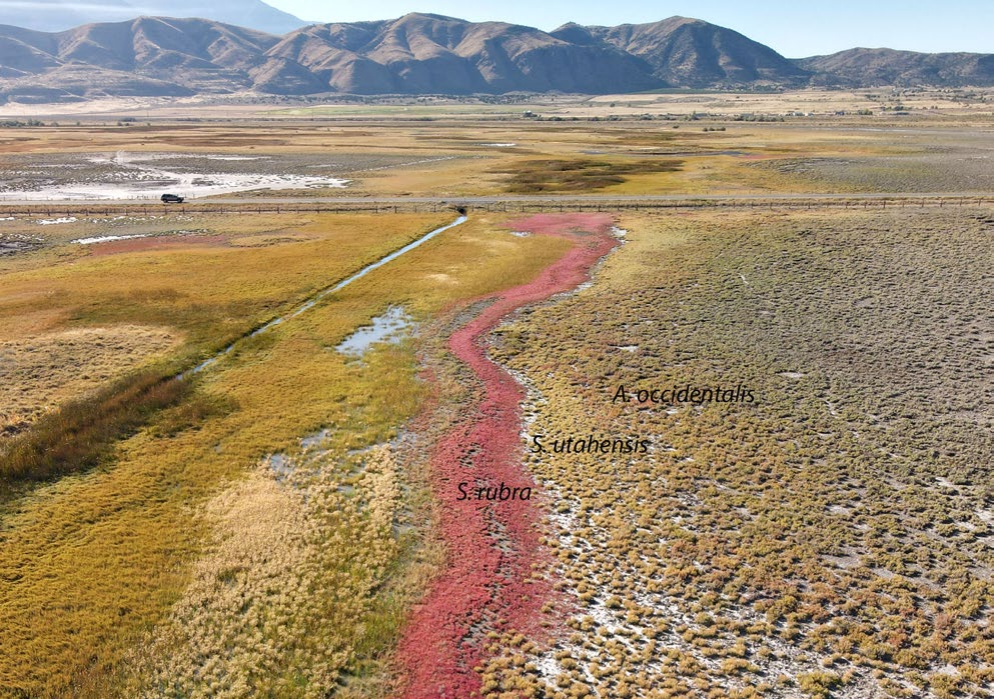
Aerial view from the north of the salt playa. The drainage canal is on the left. The vegetation zones from left to right are *Salicornia rubra*, *Sarcocornia utahensis*, *Allenrolfea occidentalis*

Our research was conducted in a salt playa approximately 2 km east of Goshen, UT, USA (39.9577983, −111.8771803). The playa contains sufficient topographic variation to result in vertical zonation of the three most important salt succulent species at the site, with *Allenrolfea occidentalis* in the highest topographic position, followed by *Sarcocornia utahensis* in the middle position, and *S*. *rubra* in the lowest position (Figure 1). The vertical distance from the top of the *S*. *rubra* zone to the bottom of the *A. occidentalis* zone is typically only 50 cm. All *S*. *rubra* plants were sampled from the *S*. *rubra* zone shown in Figure 1.

**FIGURE 2:**
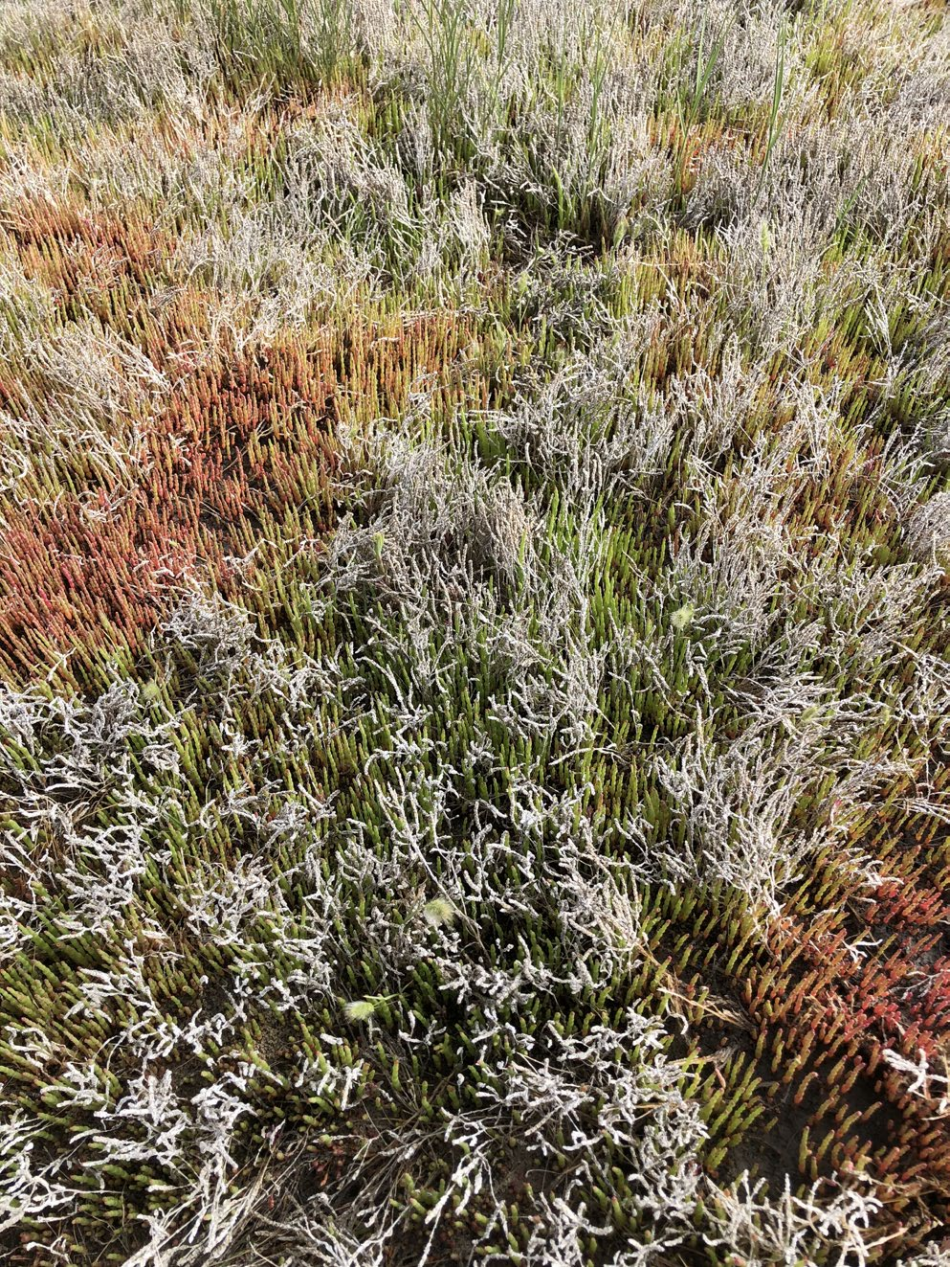
Spatial patchiness of red and green *Salicornia rubra*

On several excursions to this site, we had noted that *S*. *rubra* plants occurring in the shade of taller, dead plants from the previous year’s growth were likely to be green, whereas those receiving full sun were more likely to be red. Therefore, in May 2020 we shaded five small patches (0.5 × 0.5 m) of bright red *S*. *rubra* plants using pieces of translucent, neutral density, corrugated fiberglass panels, which blocked approximately 89% of photosynthetically active radiation (400–700 nm) as determined with a LI‐250A light meter (Li‐Cor Biosciences), and blocked approximately 99.8% of UVA + UVB radiation as determined with UVA+B digital UV radiometers (models 5.7 and 5.0; Solarmeter). Within 3 weeks of shading, formerly red plants had become bright green and were designated “Shade‐green.” Red plants within 1 m of the shaded patches and still receiving full sun were designated “Sun‐red (1).” Plants growing around the outer edges of the shades were of intermediate color; mostly green but with some red stems. These were designated “Partial shade‐interm.” Within several meters of the shade experiment, we found red plants growing in full sun, which we designated Sun‐red (2), as well as small patches of green plants growing in full sun, designated Sun‐green (Figure 2). On June 25, 2020 we harvested seven replicate samples of each of the five types of *S*. *rubra* plants [Sun‐red (1), Partial shade‐interm., Shade‐green, Sun‐red (2), Sun‐green] from the salt playa. Each sample comprised several whole individuals.

Samples were stored at 5℃ in sealed, plastic bags until they were analyzed, but in no case longer than 2 days. At the time of analysis, the shoots of each sample were rinsed in water to remove any adhering soil or salt, then dried with paper toweling. For each sample, a 0.5 g subsample was ground thoroughly with mortar and pestle to express all liquid from the cells. Ten times the weight of the sample in grams was added to the mortar as 0.4 M acetate buffer pH 4.5 to make an 11‐fold dilution of crude shoot extract. After mixing well, 2 ml of that 11‐fold dilution was centrifuged for 2 min at 10,000 *g* to remove the cellular debris as a pellet, producing a clarified extract, hereafter referred to as “extract”.

We estimated betacyanin concentrations by measuring the absorbance of the 11‐fold dilution of the extract at 540 nm (*A*_540_) (Sarker & Oba, 2019), with 0.4 M acetate buffer as the blank. The estimated betacyanin concentration was expressed as *A*_540_ g^−1^ fresh weight of extracted tissue rather than on the basis of a known betacyanin because, while the common betacyanin, betanin, is available from various chemical suppliers, it is invariably diluted with unpublished quantities of dextrin so the concentration of betanin in the final product is unknown. Following the measurement of *A*_540,_ we further diluted the 11‐fold dilution to make a 121‐fold dilution using 0.4 M acetate buffer, and quantified *A*_260_ of the 121‐fold dilution of extract as a proxy estimate of its capacity to absorb DNA‐damaging ultraviolet radiation. Again, we used 0.4 M acetate buffer as the blank. To quantify the concentration of phenolic compounds, we added Folin–Ciocâlteu reagent (TanniVer3 Tannin‐Lignin Reagent; Hach Co.) according to Hach recommendations to 121‐fold dilution of the extract and measured *A*_760_, with 0.4 M acetate buffer as the blank. Phenolics were expressed as tannic acid equivalents. All concentrations were normalized to the fresh weight of the originally extracted shoot tissue.

Absorption spectra from 250 to 850 nm were performed on 22‐fold dilutions of extracts (see above) from Sun‐red (2) and Sun‐green specimens collected August 31, 2021, using 0.4 M acetate buffer as diluent, with 0.4 M acetate buffer serving as the blank. There were no Partial shade‐interm. or Shade‐green individuals as the experiment had been previously dismantled.

We performed analyses of variance to determine whether there were significant effects of treatment on *A*_260_, *A*_540,_ and phenolic concentrations, followed by means separations using the Tukey Honestly Significant Difference method. We had originally intended to analyze the shading experiment separately from the full sun experiment. However, because the Sun‐red (1) samples in the shading experiment were never significantly different from the Sun‐red (2) samples in the full sun experiment, we analyzed all five treatments together.

A relative importance analysis was conducted using the relaimpo package (Grömping, 2021) in R (R Core Team, 2020), in which *A*_540_ of extracts, attributed to betacyanin concentration, and phenolic concentrations were used as predictors (regressors) of *A*_260_.

## 3 RESULTS

**FIGURE 3:**
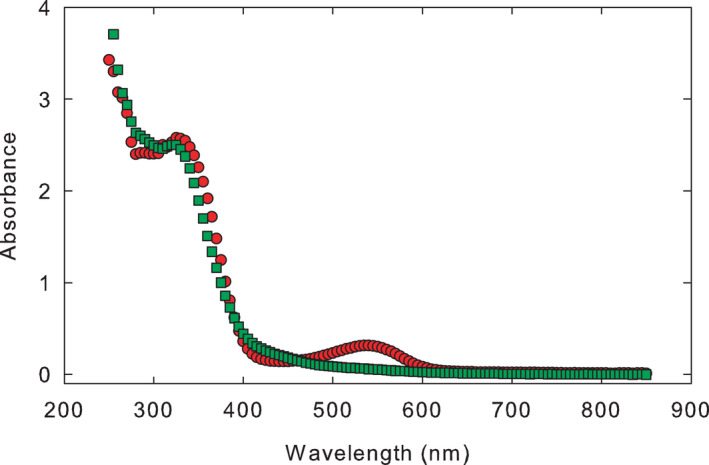
Absorbance spectra of extracts from Sun‐red (2) (circles) and Sun‐green (squares) *Salicornia rubra* plants

Typical absorption spectra for extracts from Sun‐red (2) and Sun‐green samples are given in Figure 3. The absorption spectra are essentially identical except for the broad peak around 540 nm that appears in the extract from Sun‐red (2) plants.

**FIGURE 4:**
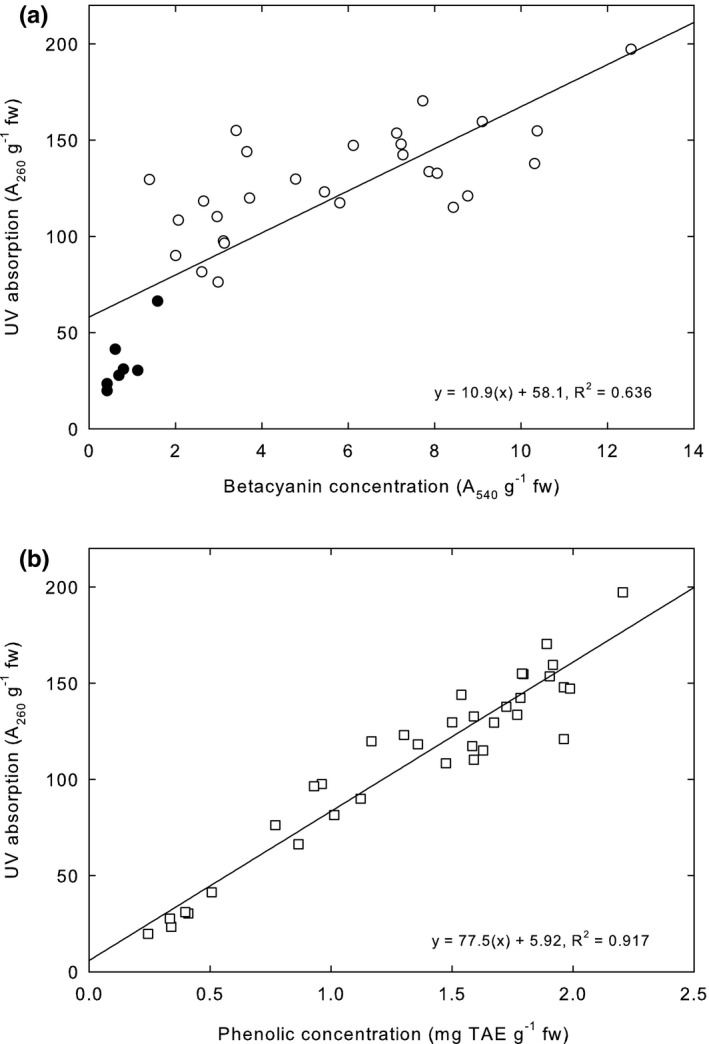
(a) The relationship between *A*_540_ of extracts, attributed to betacyanin concentration, and *A*_260_. The best fit line shown is for all data, including the plants growing in shade (filled circles). The equation for that line is given in the figure. For data not including the shaded plants (open circles), the equation for the best fit line (not shown) is *y* = 6.28(*x*) + 92.9, *R* ^2^ = .475. (b) The relationship between the concentration of total phenolics and *A*_260_. The best fit line and equation are given in the figure

Hypothesis 1 *Betacyanins absorb ultraviolet radiation*. The *A*_260_ (proxy for the capacity to absorb DNA‐damaging UV radiation) of extracts increased with *A*_540_, attributed to betacyanin concentration (Figure 4a). We, therefore, accept Hypothesis 1.

**FIGURE 5:**
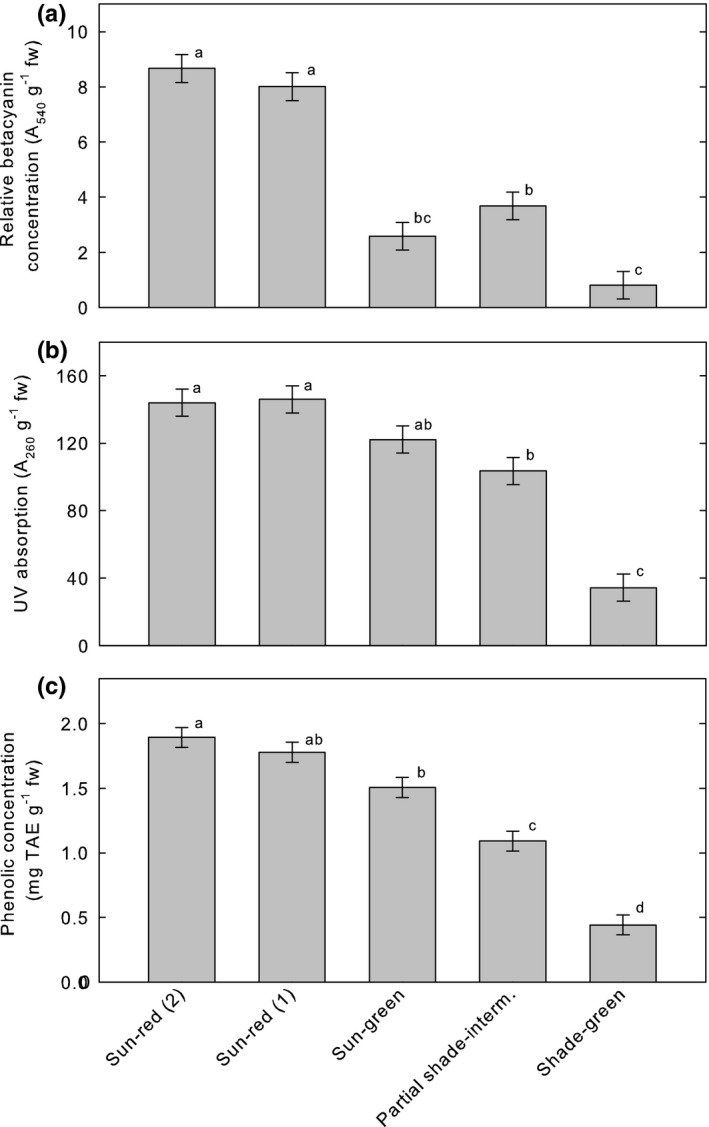
(a) Means of *A*_540_ g^−1^ fresh weight (fw) of extracts, attributed to betacyanin concentration. (b) Means of *A*_260_ g^−1^ fw. (c) Means of phenolics g^−1^ fw. See Section 2 for further explanation of plant treatments. For all panels, vertical bars are ±1 standard error of mean (SEM), means that do not share a letter are significantly different according to the Tukey Honestly Significant Difference method, and *n* = 7

Hypothesis 2a *Betacyanin concentrations are significantly greater in red plants than in green plants*. As expected, extracts from red plants [Sun‐red (2) and Sun‐red (1)] had significantly higher *A*_540_, attributed to betacyanins, than extracts from plants of intermediate color (Partial shade – interm.), and those had significantly higher *A*_540_ than extracts from green plants (Sun‐green, Shade‐green, Table S1; Figure 5a). Therefore, we accept Hypothesis 2a.

Hypothesis 2b *Betacyanin concentrations are determined by exposure to sunlight*. Extracts from plants receiving full sun [Sun‐red (2) and Sun‐red (1)] had significantly greater *A*_540_, attributed to betacyanins, than extracts from plants in the shade (Shade‐green) or partial shade (Partial shade‐interm., Figure 5a). However, extracts from green plants receiving full sun (Sun‐green) had significantly lower *A*_540_ than those from red plants receiving full sun [Sun‐red (2), Figure 5a]. Thus, color was a better predictor of betacyanin concentration than sun exposure. We reject Hypothesis 2b.

Hypothesis 3a *Absorption of ultraviolet radiation is significantly greater in red plants than in green plants*. Extracts from shade‐green plants had significantly lower *A*_260_ than those from plants of intermediate color (Partial shade‐interm.) or red plants [Sun‐red (2), Sun‐red (1), Table S2; Figure 5b]. However, extracts from green plants in full sun (Sun‐green) had significantly greater *A*_260_ than those from both green plants in the shade (Shade‐green) and plants of intermediate color (Partial shade‐interm.) (Figure 5b), indicating that color was not the primary factor determining *A*_260_. Therefore, we reject Hypothesis 3a.

Hypothesis 3b *Absorption of ultraviolet radiation is determined by exposure to sunlight*. Extracts from plants growing in full sun [Sun‐red (2), Sun‐red (1), Sun‐green] had significantly greater *A*_260_ than extracts of plants in shade (Shade‐green), while extracts of plants in partial shade (Partial shade‐interm.) exhibited an intermediate A_260_ (Tables S2; Figure 5b). Thus, the difference in the *A*_260_ between extracts from plants experiencing full sun and those from plants in the shade occurred largely irrespective of color. We accept Hypothesis 3b.

Hypothesis 4 *Compounds other than betacyanins, such as phenolic compounds, absorb significant amounts of ultraviolet radiation*. As seen in the tests of Hypothesis 2a and Hypothesis 3b, while *A*_540_ of extracts, attributed to betacyanin concentrations, were largely predictable from plant color, *A*_260_ was more directly determined by exposure to sunlight. Moreover, the concentration of phenolic compounds was significantly correlated with *A*_260_ (Figure 4b). Therefore, we accept Hypothesis 4.

Hypothesis 5a *Phenolic concentrations are significantly greater in green plants than in red plants*. In general, phenolic concentrations were significantly greater in extracts from red plants than in extracts from green plants (Tables S3; Figure 5c). We reject Hypothesis 5a.

Hypothesis 5b *Phenolic concentrations are determined by exposure to sunlight*. Phenolic concentrations were greatest in extracts from the red plants receiving full sun [Sun‐red (1) and Sun‐red (2)] and only slightly lower in extracts from green plants receiving full sun (Sun‐green) (Table S3; Figure 5c). Moreover, extracts from Sun‐green plants had significantly higher phenolic concentrations than extracts from partially shaded (Partial shade‐interm.) and fully shaded (Shade‐green) plants (Table S3; Figure 5c). We accept Hypothesis 5b.

Hypothesis 6 *Phenolic compounds are more important than betacyanins in absorbing ultraviolet radiation*. The proportion of variance explained by the two regressors (extract phenolic concentration and extract *A*_540_, attributed to betacyanin concentration) in the relative importance regression model was 91.9%, which can be apportioned between the regressors using the lmg statistic (the *R* ^2^ contribution averaged over all possible orderings of the regressors in the model, see Grömping, 2021). The lmg for phenolic concentration, a significant regressor (*p* = 6.11e^−12^), was 0.600. The lmg for *A*_540_, attributed to betacyanin concentration, not a significant regressor (*p* = .468), was 0.319. Therefore, we accept Hypothesis 6.

## 4 DISCUSSION

We set out to explore the mechanisms by which *S*. *rubra* protects itself from exposure to high levels of ultraviolet radiation. Both *A*_540_, attributed to betacyanin concentration, and phenolic concentration of extracts were correlated with *A*_260_, our proxy for the capacity to absorb DNA‐damaging ultraviolet radiation. These results suggest that both betacyanins and phenolic compounds can contribute to protection from ultraviolet radiation, as suggested by others (Del Valle et al., 2020; Sá et al., 2016). However, our relative importance analysis indicated that only phenolics contributed significantly to the capacity of the shoot extract to absorb ultraviolet radiation (*A*_260_).

The results of the relative importance analysis are consistent with our finding that exposure to sunlight was a better predictor of *A*_260_ than shoot color, and that exposure to sunlight did not have a direct impact on *A*_540_, attributed to betacyanin concentration, but did influence the concentration of phenolic compounds, which has been noted elsewhere (Del Valle et al., 2020). These results are sufficient to explain the presence of green individuals coexisting with red individuals, all in full exposure to the sun. The phenolic concentrations were not significantly higher in extracts from Sun‐green plants than from Sun‐red plants. This indicates that phenolic concentrations were not enhanced to compensate for the lack of betacyanins in Sun‐green plants, which is consistent with the idea that betacyanins do not play a large role in the absorption of ultraviolet radiation in these plants.

*Salicornia rubra* was obviously named for its red coloration. Indeed, the bright red vegetation found in salt playas of the Great Basin of the western US is frequently caused by the presence of large populations of *S*. *rubra*. We are, therefore, intrigued by the presence of the relatively small proportion of *S*. *rubra* individuals that are green in full sunlight. At our site, we have noticed that their shoots tend to be larger and more highly branched than shoots of red individuals. Because polyploidy is frequently associated with larger size in plants (Corneillie et al., 2019), we wonder whether polyploidy is somehow involved in the green coloration. Polyploidy has been observed in the genus *Salicornia* (Kadereit et al., 2007), and polyploidy can be involved in color change (McCarthy et al., 2015). The cause of being green may be a fruitful subject of future research.

In addition to absorbing ultraviolet radiation, both betacyanins (Sarker & Oba, 2019) and phenolic compounds (Aboul‐Enein et al., 2007) have the ability to counteract the effect of reactive oxygen species, which increase in concentration as a consequence of exposure to ultraviolet radiation (de Jager et al., 2017). This may explain why, when shaded, formerly red *S*. *rubra* plants become green; perhaps they have less need for antioxidants when not exposed to intense ultraviolet radiation. Unfortunately, we did not determine the relative importance of betacyanins and phenolics in terms of antioxidant capacity. That is a possible goal of future research.

## CONFLICT OF INTEREST

The authors declare no conflicts of interest.

## Supporting information

Table S1‐S3

## Data Availability

All data are available in the supporting information.
