# Supplementary material for: The protection of Salicornia rubra from ultraviolet radiation by betacyanins and phenolic compounds
Source: Plant Environ Interact. 2021 Sep 23;2(5):229–34. doi: 10.1002/pei3.10061 (PMC10168037; doi:10.1002/pei3.10061)
Supplement: Supplementary file 1 — Table S1‐S3 [file PEI3-2-229-s001.docx]

**Supporting information - Tables**

**Table S1.** Analysis of variance for A_540_ of clarified extracts, attributed to betacyanin concentration (A_540_ g^-1^ fw). The plant types include Sun-red (1), Partial shade-interm., Shade-green, Sun-red (2), Sun-green. See Materials and methods for further explanation of plant types. n=7.

| Source | DF | Adj SS | Adj MS | F | P |
| --- | --- | --- | --- | --- | --- |
| Plant type | 4 | 331.06 | 82.765 | 47.25 | <0.001 |
| Error | 30 | 52.55 | 1.752 |  |  |
| Total | 34 | 383.61 |  |  |  |

**Table S2**. Analysis of variance for A_260_ g^-1^ fw. The plant types include Sun-red (1), Partial shade-interm., Shade-green, Sun-red (2), Sun-green. See Materials and methods for further explanation of plant types. n=7.

| Source | DF | Adj SS | Adj MS | F | P |
| --- | --- | --- | --- | --- | --- |
| Plant type | 4 | 58663 | 14665.8 | 32.72 | <0.001 |
| Error | 30 | 13448 | 448.3 |  |  |
| Total | 34 | 72111 |  |  |  |

**Table S3.** Analysis of variance for phenolic concentration, tannic acid equivalents (TAE) g^-1^ fw. The plant types include Sun-red (1), Partial shade-interm., Shade-green, Sun-red (2), Sun-green. See Materials and methods for further explanation of plant types. n=7.

| Source | DF | Adj SS | Adj MS | F | P |
| --- | --- | --- | --- | --- | --- |
| Plant type | 4 | 9756327 | 2439082 | 58.42 | <0.001 |
| Error | 30 | 1252455 | 41748 |  |  |
| Total | 34 | 11008782 |  |  |  |
